# Drift-Free Foot Orientation Estimation in Running Using Wearable IMU

**DOI:** 10.3389/fbioe.2020.00065

**Published:** 2020-02-13

**Authors:** Mathieu Falbriard, Frédéric Meyer, Benoît Mariani, Grégoire P. Millet, Kamiar Aminian

**Affiliations:** ^1^Laboratory of Movement Analysis and Measurement, EPFL, Lausanne, Switzerland; ^2^Institute of Sport Sciences, University of Lausanne, Lausanne, Switzerland; ^3^Gait Up S.A., Lausanne, Switzerland

**Keywords:** running, inertial measurement units, validation study, orientation, drift, angles, foot strike

## Abstract

This study aimed to introduce and validate a new method to estimate and correct the orientation drift measured from foot-worn inertial sensors. A modified strap-down integration (MSDI) was proposed to decrease the orientation drift, which, in turn, was further compensated by estimation of the joint center acceleration (JCA) of a two-segment model of the foot. This method was designed to fit the different foot strike patterns observed in running and was validated against an optical motion-tracking system during level treadmill running at 8, 12, and 16 km/h. The sagittal and frontal plane angles obtained from the inertial sensors and the motion tracking system were compared at different moments of the ground contact phase. The results obtained from 26 runners showed that the foot orientation at mean stance was estimated with an accuracy (inter-trial median ± IQR) of 0.4 ± 3.8° and a precision (inter-trial precision median ± IQR) of 3.0 ± 1.8°. The orientation of the foot shortly before initial contact (IC) was estimated with an accuracy of 2.0 ± 5.9° and a precision of 1.6 ± 1.1°; which is more accurate than commonly used zero-velocity update methods derived from gait analysis and not explicitly designed for running. Finally, the study presented the effect initial and terminal contact (TC) detection errors have on the orientation parameters reported.

## Introduction

The orientation of the foot recorded slightly before, during, or after the ground contact phase is an essential parameter for running analysis. Many studies have investigated how different foot landing techniques give rise to kinematic and kinetic differences between subjects. For instance, the foot strike patterns (i.e., rearfoot, midfoot, and forefoot) and their association with injury risks (Cavanagh and Lafortune, 1980; Daoud et al., 2012; Goss and Gross, 2012), running economy (Perl et al., 2012; Gruber et al., 2013; Miller and Hamill, 2015; Hamill and Gruber, 2017), running performance (Larson et al., 2011; Kasmer et al., 2013; de Almeida et al., 2015), collision forces (Lieberman et al., 2010; Boyer et al., 2014; Gruber et al., 2014), muscle activity (Ahn et al., 2014; Yong et al., 2014), and footwear (Lorenz and Pontillo, 2012; Horvais and Samozino, 2013; Larson, 2014; Meyer et al., 2018) have been at the core of many research studies and changes of running paradigms within the last decades. The orientation of the foot in different planes or relative to the shank has also been extensively analyzed and now constitutes a primary marketing argument for the running industry (e.g., eversion/inversion and pronation/supination) (Nigg et al., 1993; Perry and Lafortune, 1995; Monaghan et al., 2014; Muñoz-Jimenez et al., 2015).

In research, the continuous measurement of the 3D orientation of the foot is generally obtained using optical motion capture systems (Arndt et al., 2007; Riley et al., 2008; Altman and Davis, 2012). While these systems measure the foot pose (i.e., orientation and position) accurately, they are often restricted to well-equipped laboratories and treadmill running. As an alternative to this lack of portability, a growing number of studies have shown that wearable inertial sensors, if combined with state-of-the-art algorithms, can be used to provide reliable spatiotemporal information (Camomilla et al., 2018).

Historically, the methods based on foot-worn inertial sensors that estimate the fixed-frame orientation of the foot first emerged from the field of gait analysis. Although different methods have been proposed (Sabatini et al., 2005; Mariani et al., 2010; Skog et al., 2010), most share the same underlying structure: (1) integration of the angular velocity obtained from a foot-mounted gyroscope to calculate the global frame (GF) orientation and (2) combine the measurements from other sensors (e.g., accelerometer, magnetometer, and GPS) to estimate and remove the orientation drift. Methods such as the zero-velocity-update usually require the presence of low accelerations or low magnetic disturbances during the period of stance to estimate the orientation drift. Although these periods are generally present during low-speed human locomotion, they are either rare or inexistent as the speed increases (Foxlin, 2005; Park and Suh, 2010; Zhang et al., 2017), and thus are likely to underperform in running.

Nevertheless, studies have proposed a hard reset of the drift based on the hypothetical presence of a foot-flat period during the stance phase of running (Bailey and Harle, 2014; Chew et al., 2018; Yuan et al., 2019). Although this approach seems reasonable for rearfoot strikers, it is not appropriate for forefoot strikers as their rearfoot segment possibly never comes into contact with the ground. Also, typically rearfoot strikers tend to switch from a rearfoot to a forefoot strike pattern when the running speed increases (Breine et al., 2014); speed might likewise be a confounding factor for any drift reduction method. Note that if the continuous orientation is not required, different approaches have been proposed to classify the foot-strike patterns with foot-worn inertial measurement units (IMUs) (Strohrmann et al., 2011; Giandolini et al., 2014).

The combination of strap-down integration with the difference between proximal and distal accelerations at any joint center has been used to estimate the joint orientation and to model the drift in dynamic movements (Dejnabadi et al., 2006; Fasel et al., 2018). To the authors’ knowledge, this method has never been tested to estimate the orientation drift of the foot in running.

Hence, the objective of this research was to propose a novel drift-free orientation estimation method for running built on a two-segment model of the foot and explore the abovementioned combination of proximal and distal accelerations using a single IMU placed on the rear foot. We assumed that, regardless of the foot strike pattern, a forefoot-flat period is always present, and it can be used to estimate and compensate the foot orientation drift. The proposed method provides an estimate of the orientation drift for each stance period and can, therefore, be used for online analysis of the running gait. Moreover, the proposed method does not require the presence of a second IMU on the forefoot, for such complicated instrumentation would reduce its applicability for field studies.

## Materials and Methods

### Protocol

A total of 26 volunteers (9 females and 17 males, age 29 ± 6 years, weight 70 ± 10 kg, height 174 ± 8 cm, running weekly 2.1 ± 1.0 h, 11 affiliated to a running club) participated in this study. They were running at least once a week and had no symptomatic musculoskeletal injuries. Participants gave their written informed consent before the measurements and ran for 45 s at 8, 12, and 16 km/h on a level instrumented treadmill, wearing their regular shoes. A 6 min familiarization period (Lavcanska et al., 2005) was performed on the treadmill and served as a warm-up for the participants. This protocol was approved by the local ethical committee (CCER-VD 2015-00006) and conducted according to the declaration of Helsinki.

### Wearable Systems

#### Inertial Measurement Units

**FIGURE 1:**
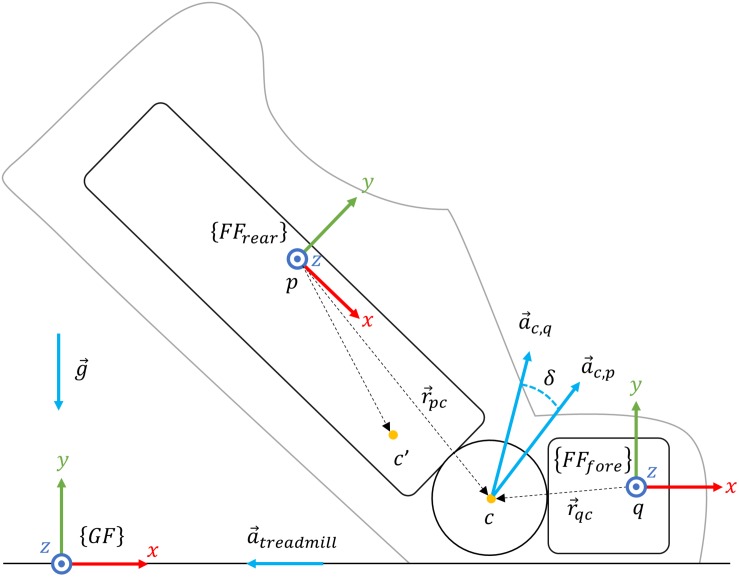
The two-segments model of the foot during the stance phase. Using the RGB convention, {*FF*_rear_} represents the FF of the rearfoot segment, {*FF*_*fore*_} the FF of the forefoot segment, and {*GF*} the room’s global frame. Points *p* and *q* are arbitrarily placed on rearfoot, and forefoot segments, *c*′ and *c* are, respectively, hypothetic and optimum rearfoot-forefoot joint’s center. ${\vec{a}}_{c,q}$ is the acceleration at c estimated from *q*, ${\vec{a}}_{c,p}$ the acceleration at *c* estimated from *p*, ${\vec{a}}_{t\,r\,e\,a\,d\,m\,i\,l\,l}$ the acceleration of the treadmill, and $\vec{g}$ the Earth gravitational acceleration. Finally, δ is the orientation difference (i.e., quaternion) between ${\vec{a}}_{c,p}$ and ${\vec{a}}_{c,q}$ while ${\vec{r}}_{p\,c}$ and ${\vec{r}}_{q\,c}$ are the distance vectors from point p to c and from q to c, respectively.

One Inertial Measurement Unit (Physilog^®^ 4, GaitUp SA, CH, weight: 19 g, size: 50 mm × 37 mm× 9.2 mm) was fixed on the dorsum of each foot using a Silicon/Velcro elastic strap. The accelerometer operated at 500 Hz (±16 g), the gyroscope at 500 Hz (±2000 deg/s), and sensors’ calibration was performed according to Ferraris et al. (1995). We modeled the foot with two rigid segments: the rearfoot and forefoot segments (Figure 1). Note that there was no sensor located on the forefoot segment. We aligned the IMU’s technical frame (TF) with the rearfoot functional frame (FF_rear_), as described by Falbriard et al. (2018); we recorded a standing period and used the gravitational acceleration to set the FF_rear_ *y*-axis parallel to the vertical axis of the foot. Then, using principal component analysis (PCA) on the running measurements, we aligned the FF_rear_ *z*-axis with the principal vector, which we assumed parallel to the mediolateral axis of the foot. Finally, we defined the FF_rear_ *x*-axis as the cross-product between the FF_rear_ *y*-axis and the *z*-axis. Note that the calibration matrix was considered constant within the duration of the trial.

#### Temporal Events Detection

Temporal events detection was based on previously validated algorithms (Falbriard et al., 2018). We segmented the trials into running strides and extracted four events per stance phase. The Initial (IC) and Terminal (TC) contact events, when the foot initializes and terminates ground contact, were found using local minima on the pitch angular velocity. Also, the mean-stance (MS) was defined as the mean time between IC and TC, and MinRot as the time-point of stance when the norm of the foot angular velocity is minimum.

#### Orientation Estimation

Strap-down integration of the angular velocity (Favre et al., 2008) is frequently used to obtain the orientation of a body segment in the GF. However, this operation generates a drift which accumulates with time. In this study, orientation estimation was performed in three phases: (i) modified strap-down integration (MSDI), (ii) drift modeling, and (iii) drift estimation and reduction. The MSDI method provides a first estimate of the orientation. It assumes that, at MinRot of each stance phase, the FF_rear_ and the GF are aligned. In other words, it supposes that a rearfoot strike is used and that, at MinRot, the rearfoot segment is flat on the ground. Since this hypothesis is not general enough (i.e., it does not consider all the possible foot strike patterns), the subsequent phases (ii) and (iii) aims to remove the drift further.

##### Modified strap-down integration

First, we set the quaternion ${}_{F\,F}^{G\,F}\,\hat{q}\,\left(t\right)$ to transform the IMU 3D kinematics from the FF_rear_ into the GF. The *x*-axis of the GF was parallel to the longitudinal axis of the treadmill’s belt, the *z*-axis to the lateral axis, and the *y*-axis was perpendicular to the ground surface, pointing upward (Figure 1). Typically, strap-down integration is computed between time-points at which the orientation of the FF_rear_ in the GF can be estimated. In walking, short zero-velocity periods during foot-flat are often used to reset the integration drift (Sabatini, 2005). As these static periods were not observed during running, we implemented a new integration method that relies on a quasi-zero velocity update at MinRot and a bidirectional strap-down integration. This method merges the strap-down integration results calculated in a forward and backward direction, awarding higher weight to the estimation originating from the closest MinRot. So, for each stride *i* ∈ [2,*N*−1], N is the total number of strides, we performed strap-down integration in two directions. The quaternion ${}_{F\,F}^{G\,F}\,{\hat{q}}_{f\,o\,r\,w\,a\,r\,d,i}\,\left(t\right)$ with *t* ∈ [MinRot(i),MinRot(i + 1)] results from the forward strap-down integration and the quaternion ${}_{F\,F}^{G\,F}\,{\hat{q}}_{b\,a\,c\,k\,w\,a\,r\,d,i}\,\left(t\right)$, with *t*∈[MinRot(i−1),MinRot(i)], from the backward integration. Note that, we assumed the FF_rear_ at MinRot [${}_{F\,F}^{G\,F}\,\hat{q}\,\left(M\,i\,n\,R\,o\,t\right)$] to be aligned with the GF. The orientation difference was obtained as in Eq. 1:

(1) q^d⁢i⁢f⁢fF⁢FG⁢F⁢(t)=q^b⁢a⁢c⁢k⁢w⁢a⁢r⁢d,i+1F⁢FG⁢F⁢(t)*q^f⁢o⁢r⁢w⁢a⁢r⁢d,iF⁢FG⁢F⁢(t)-1

We then weighted the contribution of the “backward” and “forward” estimations in the actual orientation ${}_{F\,F}^{G\,F}\,{\hat{q}}_{d\,i\,f\,f}\,\left(t\right)$ through the correction of the helical angle [α(*t*)] obtained by the transformation from the quaternion notation to the axis-angle notation (*quat2helic*):

(2) (u→⁢(t),α⁢(t))=q⁢u⁢a⁢t⁢2⁢h⁢e⁢l⁢i⁢c⁢(q^d⁢i⁢f⁢fF⁢FG⁢F⁢(t))

(3) $$\alpha_{w}\,\left(t\right)=\alpha \,\left(t\right)*\frac{t-M\,i\,n\,R\,o\,t\,\left(i\right)}{\left|t-M\,i\,n\,R\,o\,t\,\left(i+1\right)\right|}$$

The corrected helical angle α_*w*_(*t*) and vector $\vec{u}\,\left(t\right)$ were then transformed back into quaternion notation (*helic*2*quat*) and used to estimate the weighted orientation difference:

(4) q^d⁢i⁢f⁢f,wF⁢FG⁢F⁢(t)=h⁢e⁢l⁢i⁢c⁢2⁢q⁢u⁢a⁢t⁢(u→⁢(t),αw⁢(t))

Finally, we found the rearfoot orientation as:

(5) q^F⁢FG⁢F⁢(t)=q^d⁢i⁢f⁢f,wF⁢FG⁢F⁢(t)*q^f⁢o⁢r⁢w⁢a⁢r⁢d,iF⁢FG⁢F⁢(t)

Since the forward and backward orientation estimations are linearly weighted (Eq. 3), this technique does not have jumps in the final orientation estimate ${}_{F\,F}^{G\,F}\,\hat{q}\,\left(t\right)$.

##### Drift modeling based on joint center acceleration

During the stance phase of running, the kinematics of the rearfoot and forefoot segments vary upon the landing technique. The forefoot segment always has a short flat period, independently of the foot strike pattern (i.e., rearfoot strike, midfoot strike, or forefoot strike), while the rearfoot segment is usually flat only for rearfoot strikes. However, all runners have the forefoot segment that remains flat on the ground shortly after toe-strike and toward most of the pushing phase (Cavanagh and Lafortune, 1980; De Cock et al., 2005). The previously calculated rearfoot orientation ${}_{F\,F}^{G\,F}\,\hat{q}\,\left(t\right)$ could, therefore, be incorrect due to this potential absence of the rearfoot-flat period. By modeling the foot with two segments, one can estimate the acceleration at their joint center (i.e., point c in Figure 1) based on the rearfoot [${\vec{a}}_{c,p}\,\left(t\right)$] and forefoot [${\vec{a}}_{c,q}\,\left(t\right)$] accelerations. The above can be done using the function $\varphi \,\left(\vec{a},\vec{\omega },\vec{r}\right)$ which shifts the acceleration $\vec{a}\,\left(t\right)$ of any point of a segment to another point of the same segment based on the segment’s angular velocity $\vec{\omega }\,\left(t\right)$ and the translation between the two points $\vec{r}$:

(6) $$\varphi \,\left(\vec{a}\,\left(t\right),\vec{\omega }\,\left(t\right),\vec{r}\right)=\vec{a}\,\left(t\right)+\vec{\dot{\omega }}\,\left(t\right)\times \vec{r}+\vec{\omega }\,\left(t\right)\times \left(\vec{\omega }\,\left(t\right)\times \vec{r}\right)$$

The drift model in this study assumes that, during the forefoot-flat period, ${\vec{a}}_{c,p}\,\left(t\right)-{\vec{a}}_{c,q}\,\left(t\right)=\vec{0}$. Consequently, the orientation difference of the joint center accelerations (JCAs) [δ(t)] should also be zero or minimal. During forefoot-flat, ${\vec{a}}_{c,q}\,\left(t\right)$ can be estimated from Eq. 6 by assuming no angular rotation:

(7) $${\vec{a}}_{c,q}\,\left(t\right)={\vec{a}}_{q}\,\left(t\right)=\vec{g}+{\vec{a}}_{t\,r\,e\,a\,d\,m\,i\,l\,l}\,\left(t\right)$$

where $\vec{g}$ is the earth gravitational acceleration and ${\vec{a}}_{t\,r\,e\,a\,d\,m\,i\,l\,l}\,\left(t\right)$ the acceleration of the treadmill. Note that, even if the treadmill velocity was set constant, the shearing forces acting on the belt, shortly after landing, change the speed of the treadmill, hence generating a non-zero acceleration ${\vec{a}}_{t\,r\,e\,a\,d\,m\,i\,l\,l}\,\left(t\right)$. The model also assumes that each point on the rearfoot segment has a trajectory which lies on the surface of a sphere during forefoot-flat; hence, Eq. 8 describes the accelerations acting at point p:

(8) $${\vec{a}}_{p}\,\left(t\right)={\vec{a}}_{p,t\,a\,n\,g}\,\left(t\right)+{\vec{a}}_{p,c\,e\,n\,t}\,\left(t\right)+\vec{g}+{\vec{a}}_{t\,r\,e\,a\,d\,m\,i\,l\,l}\,\left(t\right)$$

Where, ${\vec{a}}_{p,t\,a\,n\,g}\,\left(t\right)$ is the tangential and ${\vec{a}}_{p,c\,e\,n\,t}\,\left(t\right)$ the centripetal acceleration at point *p*.

##### Drift estimation and reduction

To estimate the orientation drift δ(t), the accelerations ${\vec{a}}_{c,p}\,\left(t\right)$ and ${\vec{a}}_{c,q}\,\left(t\right)$ were calculated based on the acceleration and angular velocity at point p and q, respectively. As the exact position (*p*) of the IMU is unknown (i.e., somewhere on the dorsum of the foot), we designed a two-step optimization process to find the ${\vec{r}}_{p\,c}$ vector, necessary to find ${\vec{a}}_{c,p}\,\left(t\right)$. In the first step, the point *c*′ is selected as the candidate position, which minimizes the norm of the tangential and centripetal accelerations. This point *c*′ is chosen among all the rearfoot points *j* for which *r*_*pj,x*_ < 30 cm, *r*_*pj,y*_ < 10 cm and *r*_*pj,z*_ < 5 cm in FF_rear_. We used Eq. 6 to find an estimate of ${\vec{r}}_{p\,c}$, namely ${\vec{r}}_{p\,c^{\prime }}$, which minimizes Eq. 9 after removing the contribution of $\vec{g}$ (inclination) from the acceleration at point *j*:

(9) r→p⁢c′=argminr→p⁢j(||φ⁢(a→p⁢(t)-q^F⁢FG⁢F⁢(t)-1*G⁢Fg→,ω→p⁢(t),r→p⁢j)||),t∈S

The function argmin returns the ${\vec{r}}_{p\,j}$ vector at which the input function is minimized. *S* is the set of samples within the pushing phase of each stride i, defined as *t* ∈ [*MinRot*(*i*),*TC*(*i*)]. During the pushing phase, the tangential and centripetal accelerations are maximum. This high signal-to-noise ratio optimizes the outcome of the minimalization function in Eq. 9. As a result, Eq. 6 and ${\vec{r}}_{pc{}^{\prime }}$ can be used to estimate the acceleration at the point *c*′:

(10) $${\vec{a}}_{c^{\prime },p}\,\left(t\right)=\varphi \,\left({\vec{a}}_{p}\,\left(t\right),{\vec{\omega }}_{p}\,\left(t\right),{\vec{r}}_{p\,c^{\prime }}\right)$$

If the point *c*′ is reasonably close to the joint center *c*, the tangential and centripetal accelerations should approximately be null and, based on Eq. 7, ${\vec{a}}_{c^{\prime },p}\,\left(t\right)-\vec{g}$ can be used as an estimate of ${\vec{a}}_{t\,r\,e\,a\,d\,m\,i\,l\,l}\,\left(t\right)$.

(11) $${\vec{a}}_{c^{\prime },p}\,\left(t\right)≅{\vec{a}}_{c,q}\,\left(t\right)=\vec{g}+{\vec{a}}_{t\,r\,e\,a\,d\,m\,i\,l\,l}\,\left(t\right)$$

(12) $${\vec{a}}_{c^{\prime },p}\,\left(t\right)-\vec{g}≅{\vec{a}}_{t\,r\,e\,a\,d\,m\,i\,l\,l}\,\left(t\right)$$

In the second step of optimization, ${\vec{a}}_{c^{\prime },p}\,\left(t\right)$ was used as in Eq. 9 to refine the estimate of ${\vec{r}}_{p\,c}$:

(13) $${\vec{r}}_{p\,c}=\underset{{\vec{r}}_{p\,x}}{\text{argmin}}||\varphi \,\left({\vec{a}}_{p}\,\left(t\right)-{\vec{a}}_{c^{\prime },p}\,\left(t\right),{\vec{\omega }}_{p}\,\left(t\right),{\vec{r}}_{p\,x}\right)||$$

(14) $${\vec{a}}_{c,p}\,\left(t\right)=\varphi \,\left({\vec{a}}_{p}\,\left(t\right),{\vec{\omega }}_{p}\,\left(t\right),{\vec{r}}_{p\,c}\right)$$

Using Eq. 7 and Eq. 14 in GF, the orientation drift [δ(t)] was estimated, for each step, as the orientation difference between ${\vec{a}}_{c,p}\,\left(t\right)$ and ${\vec{a}}_{c,q}\,\left(t\right)$:

(15) $$\delta \,\left(\text{t}\right)=\left[c\,o\,s\,\left(\frac{\beta \,\left(t\right)}{2}\right),\sin\left(\frac{\beta \,\left(t\right)}{2}\right)*v\,\left(t\right)\right]$$

where *v*(*t*) is a unit vector perpendicular to ${\vec{a}}_{c,p}\,\left(t\right)$ and ${\vec{a}}_{c,q}\,\left(t\right)$ and β(*t*) is the rotation around *v*(*t*):

(16) $$\beta \,\left(t\right)=a\,c\,o\,s\,\left(\frac{{\vec{a}}_{c,q}\,\left(t\right)*{\vec{a}}_{c,p}\,\left(t\right)}{\left|{\vec{a}}_{c,q}\,\left(t\right)\right|*\left|{\vec{a}}_{c,p}\,\left(t\right)\right|}\right)$$

(17) $$v\,\left(t\right)=\frac{{\vec{a}}_{c,q}\,\left(t\right)\times {\vec{a}}_{c,p}\,\left(t\right)}{\left|{\vec{a}}_{c,q}\,\left(t\right)\times {\vec{a}}_{c,p}\,\left(t\right)\right|}$$

The orientation drift of the ith stance phase, namely δ_*i*_, was defined as the average quaternion (Markley et al., 2007) of δ(t) where t ∈ [t_*m*_−ε, t_*m*_ + ε]. The parameter t_*m*_ was found as in Eq. 18 and ε = 5 ms.

(18) $$t_{m}=\min_{t}\left(||{\vec{a}}_{c,p}\,\left(t\right)||-1\right)$$

(19) $$\delta_{i}=m\,e\,a\,n\,\left(\delta \,\left(t\right)\right),t\in \left[t_{m}-\varepsilon ,\,t_{m}+\varepsilon \right]$$

We then estimated the rearfoot orientation based on Eq. 5 and obtained the drift correction with Eq. 20:

(20) qF⁢FG⁢F⁢(tm)=q^F⁢FG⁢F⁢(tm)*k*δi

(21) $$k=1/\left(1+e^{100*\left(||{\vec{a}}_{c,p}\,\left(t_{m}\right)||-1.1\right)}\right)$$

Note that the sigmoid function in Eq. 21 aims to reduce the weight of the update the further the norm of ${\vec{a}}_{c,p}\,\left(t_{m}\right)$ is from the unit norm. The parameters of the sigmoid function were selected such that a 10% error corresponds to a coefficient k equal to 0.5. Finally, we used the same process as the MSDI method to correct the estimate of ${}_{F\,F}^{G\,F}\,\hat{q}\,\left(t\right)$: for each stance phase a new ${}_{F\,F}^{G\,F}\,{\hat{q}}_{f\,o\,r\,w\,a\,r\,d,i}$ and ${}_{F\,F}^{G\,F}\,{\hat{q}}_{b\,a\,c\,k\,w\,a\,r\,d,i}$ was computed, with the center time t*_*m,i*_* and with an initial orientation defined as in Eq. 19.

### Reference System

#### Temporal Events Detection

We used an instrumented treadmill (T-170-FMT, Arsalis, Belgium) as a reference system for temporal events detection. The force plate (FP) recorded the 3D ground reaction forces (GRF) at 1000 Hz, and a 5 V analog trigger synchronized the system with the IMUs. To reduce the noise on the vertical GRF signal due to the treadmill’s vibration, we first applied a 2nd-order stopband Butterworth filter with edge frequencies set to 25 and 65 Hz. Finally, the initial and terminal contact (TC) events were found using a threshold on the vertical GRF set at 7% of the participant’s body weight (Falbriard et al., 2018).

#### The 3D Orientation of the Foot With Stereophotogrammetry

Motion tracking of the lower limbs was achieved using eight motion cameras (BTS Smart 400, BTS Bioengineering, United States) and 21 reflective markers placed on body landmarks. The system operated at 100 Hz and was synchronized with the IMUs and the FPusing an analog trigger (i.e., 5 V pulse trigger recorded on all the systems). We defined the GF of the system using three reflective markers on the horizontal plane of the treadmill; the GF *x*-axis set parallel to the belt (i.e., in the running direction) and the *z*-axis laterally to the belt, and the *y*-axis perpendicular to the *x* and *z* axis (Figure 1).

##### Calibration in standing posture

**FIGURE 2:**
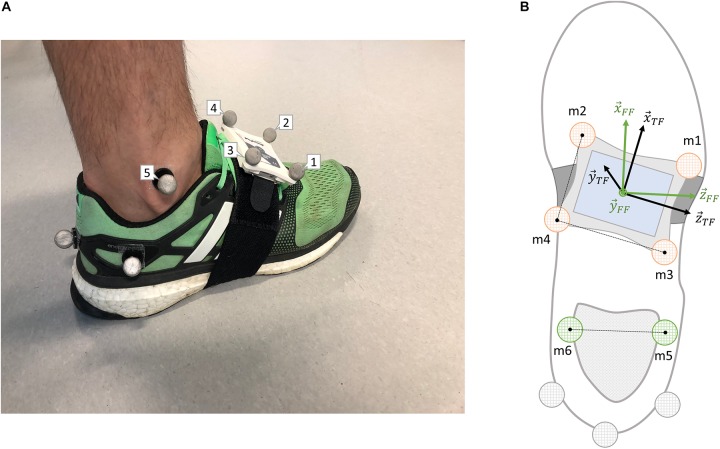
**(A)** Rear/lateral view of the markers’ configuration used in this study. **(B)** Top scheme of the markers’ configuration required in the definition of the foot’s technical (TF) and functional (FF) frames. Markers illustrated in orange are the one needed to set the TF, in green the FF, and in gray the duplicates which were not used in this study. Also, note that markers 5 and 6 were kept only during the calibration trials.

The malleolus markers (Figure 2, m5–6) were frequently torn off while running, so we recorded a 5-s standing posture at the beginning of each session. The calibration phase aimed to obtain the matrix ${}_{T\,F}^{F\,F}\,R_{c\,a\,l\,i\,b}$ which transforms the vector space from the TF to the FF. The position of the markers of the shoe remained unchanged throughout a session, so the matrix ${}_{T\,F}^{F\,F}\,R_{c\,a\,l\,i\,b}$ was considered constant and was used to process the running trials. The TF was defined using the mean position of 4 reflective markers firmly fixed around the IMU (Figure 2, m1–4) and the matrix ${}_{T\,F}^{G\,F}\,R_{c\,a\,l\,i\,b}$ was set to transform the vector space from the TF to the GF (Eqs. 22 and 23). In Eq. 22, the 𝒩 symbol represents the normalization function, and the circumflex indicates that the average position of the marker was considered.

(22) $$\begin{array}{c} {\vec{\tilde{x}}}_{T\,F}=𝒩\,\left({\vec{\hat{m}}}_{2}-{\vec{\hat{m}}}_{4}\right),{\vec{z}}_{T\,F}=𝒩\,\left({\vec{\hat{m}}}_{3}-{\vec{\hat{m}}}_{4}\right),\\ {\vec{y}}_{T\,F}=𝒩\,\left({\vec{z}}_{T\,F}\times {\vec{\tilde{x}}}_{T\,F}\right),{\vec{x}}_{T\,F}={\vec{y}}_{T\,F}\times {\vec{z}}_{T\,F} \end{array}$$

(23) Rc⁢a⁢l⁢i⁢bT⁢FG⁢F=[x→T⁢F,y→T⁢F,z→T⁢F]

We defined the orientation of the FF (${}_{F\,F}^{G\,F}\,R_{c\,a\,l\,i\,b}$) using the two malleolus markers (m5-6) and the GF vertical axis, as shown in Eqs. 24 and 25.

(24) $$\begin{array}{c} {\vec{\tilde{z}}}_{F\,F}=𝒩\,\left({\vec{\hat{m}}}_{8}-{\vec{\hat{m}}}_{9}\right),{\vec{y}}_{F\,F}=\left[0,1,0\right],\\ {\vec{x}}_{F\,F}=𝒩\,\left({\vec{y}}_{F\,F}\times {\vec{\tilde{z}}}_{F\,F}\right),{\vec{z}}_{F\,F}={\vec{x}}_{F\,F}\times {\vec{y}}_{F\,F} \end{array}$$

(25) Rc⁢a⁢l⁢i⁢bF⁢FG⁢F=[x→F⁢F,y→F⁢F,z→F⁢F]

Finally, we obtained the matrix ${}_{T\,F}^{F\,F}\,R_{c\,a\,l\,i\,b}$ using the two calibration matrices from Eq. 23 and Eq. 25.

(26) Rc⁢a⁢l⁢i⁢bT⁢FF⁢F=Rc⁢a⁢l⁢i⁢b′F⁢FG⁢F*Rc⁢a⁢l⁢i⁢bT⁢FG⁢F

##### Reference orientation during running

During the running trials, only the markers m1 to m4 were kept. We calculated the TF of the foot as in Eq. 22 except that the markers’ position at each time *t* was considered and not their average position as for the calibration trials.

(27) RT⁢FG⁢F⁢(t)=[x→T⁢F⁢(t),y→T⁢F⁢(t),z→T⁢F⁢(t)]

Finally, we transformed the TF into the FF using the matrix from the calibration trial.

(28) RF⁢FG⁢F⁢(t)=(Rc⁢a⁢l⁢i⁢bT⁢FF⁢F*RT⁢FG⁢F⁢(t)′)′=RT⁢FG⁢F⁢(t)*Rc⁢a⁢l⁢i⁢b′T⁢FF⁢F

By definition, the columns of ${}_{F\,F}^{G\,F}\,R\,\left(t\right)$ correspond to the coordinates of the TF basis vectors in the GF. The TF was computed based on the markers affixed on the IMU and was, therefore, subject to fixation artifact. Three additional markers were placed on the subtalar region as duplicates in case of unsatisfactory data quality. These markers were fixed on the shoe but suffered from recurrent marker loss as the marker on the medial side was frequently hit by the opposite foot during running. When present, however, these markers were used to visually assess the sensor-to-foot motion (i.e., wobbling of the sensor) with an average RMS difference of 3.68° obtained after the low-pass filtering of the pitch angle.

### Validated Angles

We calculate two reference angles using the 3D orientation of the foot measured by stereophotogrammetry [${}_{F\,F}^{G\,F}\,R\,\left(t\right)$]: the pitch angle (θ_ref_), defined as the projection of the FF *x*-axis onto the sagittal plane in the GF and the roll angle (ρ_ref_), defined as the projection of FF *z*-axis onto the frontal plane in the GF. These angles were also computed for the IMU system using the MSDI [${}_{F\,F}^{G\,F}\,\hat{q}\,\left(t\right)$] method (θ_MSDI_, ρ_MSDI_) and the JCA [qF⁢FG⁢F(t)] method (θ_JCA_, ρ_JCA_). By definition, the pitch angle is zero when the rear foot remains flat on the ground and is positive when the forefoot segment is higher than the rearfoot segment. The root mean square error (RMSE) of θ_ref_(stance)–θ_MSDI_(stance) and θ_ref_(stance)–θ_JCA_(stance) was estimated for each stance phase. In addition, the value of the pitch angle at initial contact (IC), i.e., the foot strike angle [θ_ref_(IC), θ_MSDI_(IC), and θ_JCA_(IC)], at terminal contact (TC), i.e., the pushing angle [θ_ref_(TC), θ_MSDI_(TC), and θ_JCA_(TC)] and at mean-stance [θ_ref_(MS), θ_MSDI_(MS), and θ_JCA_(MS)] were extracted from the different methods. These parameters rest on the detection accuracy of the IC and TC, so they were computed based on the results of both the FP (i.e., reference system) and the IMU (Falbriard et al., 2018). Note that, because of the potential detection error of the IMU-based method, we used the mean angle within an 8-millisecond window (i.e., ±2 samples at 500 Hz) instead of the exact angle at IC, MS, and TC. Moreover, the time (AC) and value of the pitch angle last local maximum before IC was extracted and defined as the pre-activation pitch angle [θ_ref_(AC), θ_MSDI_(AC), and θ_JCA_(AC)]. This feature describes the orientation of the foot shortly before landing when muscle pre-activation occurs (Kyröläinen et al., 2005). Also, as the range of the roll angle was small, therefore potentially suffering from low signal-to-noise ratio, only the activation roll angle [ρ_ref_(AC), ρ_MSDI_(AC), and ρ_JCA_(AC)] was defined as the last local minimum before IC. A negative roll angle corresponds to an inversion of the foot and a positive angle to an eversion.

### Statistical Analysis and Error Computation

This study focused on the trials at 8, 12, and 16 km/h, which corresponds to slow, moderate, and fast running. Trials were either removed because of instrumentation errors, protocol errors, or marker loss. We also removed the outliers steps from the data set according to the following criteria: θ_ref_(MS) >10 and θ_ref_(AC) <-80. After the outliers were removed, trials with less than five strides were dropped from the study.

To evaluate the error of the IMU estimations against the reference motion tracking system, we computed four statistics on the entire data set for each parameter. The bias (intra-trial mean) and precision (intra-trial STD) were computed on the strides from the same foot and the same trial. We considered the feet independently as runners may use different patterns for the left and right foot. The bias and precision were later combined among all the trials: b_μ_ the inter-trials median of the bias, b_σ_ the inter-trials IQR of the bias, σ_μ_ the inter-trials median of the precision and σ_σ_ the inter-trials IQR of the precision. We used the median and IQR statistics because the biases and precisions were not normally distributed.

The influence of the running speed on the intra-trial biases and precision values was tested using the non-parametric Kruskal–Wallis test with a significance level set at *p* < 0.1. This test was preferred to an ANOVA analysis because of the low number of trials and the lack of prior knowledge about the seemingly not normal distributions. Also, boxplots were used to visualize the biases and precision differences among the running speeds. Finally, we graphically assessed the agreement between the IMU-based system and the reference motion capture system using Bland–Altman plots (Bland and Altman, 1986).

## Results

In total, 4252 steps were analyzed in this study. The mean ± STD (min, max) number of recorded strides per trial was 36 ± 6 (10, 45) for a total of 59 trials (23 at 8 km/h, 23 at 12 km/h, and 13 at 16 km/h).

**FIGURE 3:**
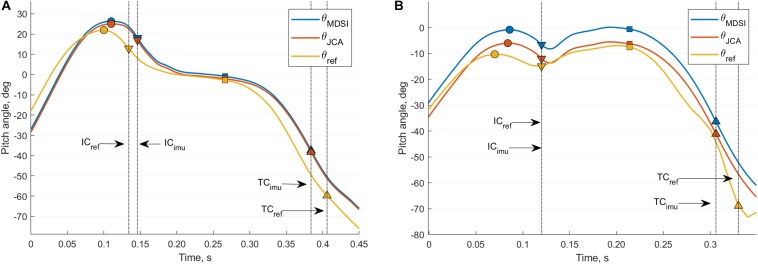
Comparison of the pitch angle measured from different measurement systems for a rearfoot **(A)** and forefoot **(B)** striker. The blue curve is the estimation from the IMU-based MSDI method (θ_MSDI_), the orange curve from the IMU-based JCA method (θ_JCA_), and the yellow curve from the reference motion tracking system (θ_ref_). The IC events are shown using down-pointing triangles, TC events with up-pointing triangles, MS events with squares, and the AC peaks using circles. The black vertical dashed lines accentuate the detection differences, for the IC and TC events, between the IMU and the FP system.

The pitch angles during the stance phase and obtained from the different estimation methods are shown for a rearfoot (left) and a forefoot (right) striker in Figure 3. We emphasized on the IC and TC detection differences between the FP and the IMU-based method using vertical dashed lines.

Table 1 shows the results of the inter-trials error statistics for the MSDI and JCA orientation estimation methods. The range (95% interval) observed on the reference system for the pitch and roll angles are: θ_ref_(IC) (−12.5, 18.2), θ_ref_(MS) (−11.8, −1.2), θ_ref_(TC) (−68.9, −41.6), θ_ref_(AC) (−7.8, 28.9), and ρ_ref_(AC) (−29.5, −7.6). The error statistics are expressed in degrees and are shown for two different temporal events detection systems: the IMUs and the FP. When the steps are gathered regardless of their trial and using the IMU-based event detection, the mean ± STD error (°) of the JCA method are: θ_JCA_(IC) (0.8 ± 5.9), θ_JCA_(MS) (0.2 ± 4.7), θ_JCA_(TC) (17.0 ± 9.0), θ_JCA_(AC) (2.1 ± 5.5), and ρ_JCA_(AC) (3.1 ± 4.9). Similarly, for the MSDI method: θ_MSDI_(IC) (3.9 ± 5.7), θ_MSDI_(MS) (3.6 ± 3.9), θ_MSDI_(TC) (20.2 ± 8.8), θ_MSDI_(AC) (5.3 ± 5.2), and ρ_MSDI_(AC) (4.6 ± 4.9).

**TABLE 1:** Inter-trial analysis of the IMU-based pitch (θ) and roll (ρ) angles estimation errors with motion tracking cameras used as a reference.

**Parameter**	**IMU-Based event detection**				**FP-Based event detection**			
	**Bias (°)**		**Precision (°)**		**Bias (°)**		**Precision (°)**	
	**b_µ_**	**b_σ_**	**σ_µ_**	**σ_σ_**	**b_µ_**	**b_σ_**	**σ_µ_**	**σ_σ_**
θ_JCA_(IC)	0.9	6.7	2.4	1.5	6.2	6.9	2.2	1.1
θ_MSDI_(IC)	3.7	6.0	2.1	1.1	9.9	7.2	1.8	0.8
θ_JCA_(IC)−θ_MSDI_(IC)	–2.9	0.7	0.3	0.4	–3.7	–0.3	0.3	0.3
θ_JCA_(MS)	0.4	3.8	3.0	1.8	1.1	3.5	1.3	1.1
θ_MSDI_(MS)	3.8	3.8	2.0	1.7	4.3	3.1	0.8	0.4
θ_JCA_(MS)−θ_MSDI_(MS)	–3.5	0.0	0.9	0.1	–3.2	0.4	0.5	0.8
θ_JCA_(TC)	17.4	8.7	3.2	1.7	2.6	8.0	2.8	1.5
θ_MSDI_(TC)	20.8	7.6	2.8	1.3	4.8	7.1	2.4	1.4
θ_JCA_(TC)−θ_MSDI_(TC)	–3.4	1.1	0.3	0.4	–2.2	0.9	0.4	0.1
θ_JCA_(AC)	2.0	5.9	1.6	1.1				
θ_MSDI_(AC)	5.0	5.7	1.2	0.9				
θ_JCA_(AC)−θ_MSDI_(AC)	–3.0	0.2	0.3	0.2				
θ_JCA_(stance)	4.5	2.8	0.8	0.6				
θ_MSDI_(stance)	6.2	4.1	0.6	0.3				
θ_JCA_(stance)−θ_MSDI_(stance)	–1.7	–1.3	0.2	0.3				
ρ_JCA_(AC)	3.0	5.7	1.5	1.3				
ρ_MSDI_(AC)	4.4	5.5	1.5	1.2				
ρ_JCA_(AC)−ρ_MSDI_(AC)	–1.4	0.2	0.0	0.1				

*The MSDI and JCA methods are evaluated in two scenarios: (1) Using temporal event detection from the IMU. (2) Using temporal event detection from the force plate (FP). The selected events are: IC, initial contact; MS, mean-stance; TC, terminal contact; AC, activation peak. The results of the inter-trials median (b*_μ_*) and IQR (b*_σ_*) of the bias, the inter-trials median (σ*_μ_*) and IQR (σ*_σ_*) of the precision and the root mean square error (RMSE) of the continuous pitch angle [θ*_JCA_*(stance), θ*_MSDI_*(stance)] between IC and TC are presented in this table.*

**FIGURE 4:**
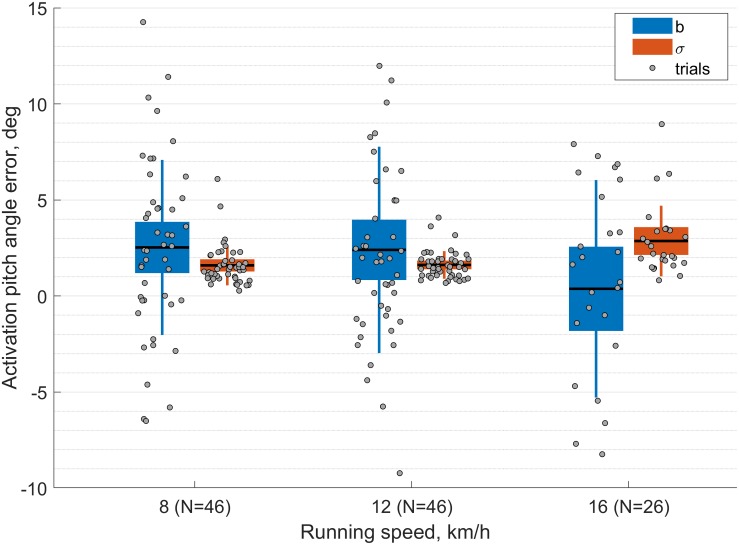
Boxplot of the intra-trial biases and precision results for the foot pitch activation angle [θ_JCA_(AC)] measured with the proposed method (JCA). In the figure, the intra-trial biases are shown in blue and the precision values in orange. The gray dots represent the statistic of each trial. Note that there are two dots per trial because the feet were considered independently.

Figure 4 illustrates the intra-trial biases (b) and precision (σ) statistics obtained for the θ_JCA_(AC) parameter at 8, 12, and 16 km/h. Two trials at 8 km/h had large biases (23.2° and −14.8°) and were cut-off from the graph for the sake of illustration. The results from the Kruskal-Wallis test suggest that the biases (*b*) and precision (σ) values for θ_JCA_(AC) and θ_JCA_(MS) are not affected by the running speed. However, for the parameters θ_JCA_(IC), θ_JCA_(TC) and ρ_JCA_(AC) the precision (σ) of the system was significantly affected (*p* < 0.05) but the intra-trial biases (b) were not (*p* = 0.11, *p* = 0.21, *p* = 0.42).

**FIGURE 5:**
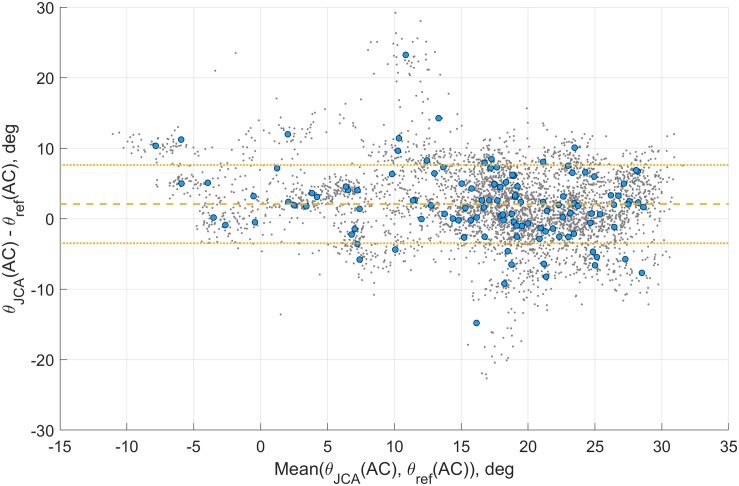
Bland–Altman plot of the activation pitch angle [θ_JCA_(AC)] for the JCA method. The gray dots show the agreement of each step, the blue circles the agreement of the intra-trial mean, and the yellow lines the mean ± STD of the error.

Finally, a Bland–Altman plot (Figure 5) shows the agreement between the IMU-based system and the reference motion capture system, with the mean (2.1) ± STD (5.2) of the error displayed with yellow horizontal lines.

## Discussion

In this study, we proposed a new method to estimate the foot orientation during running, based on a single IMU on the rear foot. While IMU-based estimation generates drift due to strap-down integration operation, we proposed a MSDI supplemented with a drift compensation method (JCA). The technique relies on the assumption of a flat period in the forefoot, which is accurate for all landing strike pattern. Assuming dynamic rearfoot kinematics during the pushing phase and a static period on the forefoot segment, a two-segment biomechanical model of the foot is, therefore, well suited for running. Also, the system requires no prior knowledge about the sensor’s location. The IMU must be fixed on rearfoot, and the functional calibration is automatically performed. Validated against gold standard stereophotogrammetry system, the proposed drift correction method allowed to estimate the foot orientation at mean stance phase with a bias of 0.4 ± 3.8° and precision of 3.0 ± 1.8°. Note that the validation was restricted to the stance phase due to the occlusion of the markers’ position during the swing phase.

In this study, the drift correction of the MSDI method is hypothetical and enforced. An implication of this is the possibility that the biases (b_µ_ and b_σ_) observed for this method cannot be extrapolated to other populations or other running conditions (e.g., various speed and style); we would certainly expect more significant biases in the case of forefoot strikers (Figure 3B). Instead, the JCA method uses a real measure of the orientation drift and correctly estimated the pitch angle around MS, while the MSDI method remained around 0° (Figure 3). The RMSE analysis (Table 1) also reveals better results for the JCA method with better accuracy (b_µ_ and b_σ_ statistics). In contrary, the σ_µ_ and σ_σ_ statistics suggest that the JCA method is slightly less precise. Note that these precision differences are always below 1 (Table 1) and seem reasonable given that the θ_MSDI_(MS) precision exposes the inter-steps variability of the participants (i.e., of the reference system). Besides, the precision of the JCA system could be improved by tuning the parameters of the sigmoid function of Eq. 21 in order to reduce the effect of outlier estimations. Also, the algorithm provides near-real-time processing of the orientation (i.e., in the order of a step), and could potentially be improved by considering the orientation of the few preceding steps in the estimation of the drift (e.g., using a weighted average).

Table 1 and Figure 3 highlight the importance of temporal events detection accuracy in the estimation of the pitch angle at IC, MS, and TC. Large errors in the measured angles result from the fact that IC and TC events are detected during phases of rapid change in pitch angle. Table 1 reveals that, when the FP system detected TC, the median bias (b_µ_) of the JCA and the MSDI methods improved by 14.8° and 16°, respectively. Similarly, the biases were worsened by 5.3° and 6.2° for IC. These findings can be explained by the fast-changing slope of the pitch angle (θ) around the IC, while it is continuously negative around TC. In consequence, the detection biases of IC and TC have dissimilar effects on θ_JCA_(IC), θ_MSDI_(IC), θ_JCA_(TC), and θ_MSDI_(TC). Furthermore, these estimations are sensitive to synchronization delays between the reference systems and the IMUs.

Because the AC event is not affected by the temporal events detection technique, a more detailed analysis was performed on the θ_JCA_(AC) parameter. The parameter ρ_JCA_(AC) is also unaffected by the detection accuracy of the temporal events; however, the pitch angle was preferred because of the lack of generality in the roll angle drift correction hypothesis. The assumption of a null roll angle for the forefoot segment may be incorrect for subjects with pathological pronation/supination. In Figure 5, the optical motion-tracking system and the IMU-based system demonstrate a good agreement across the range of angles.

Bailey and Harle (2014) used two methods (linear de-drifting and extended Kalman filter) to compute the orientation of the foot in 5 subjects based on shoe-mounted IMUs. They reported an error (mean + STD) for θ(IC) of 1.92 ± 1.09° at 8.28 km/h and 3.18 ± 1.19° at 12.24 km/h. The present JCA results (0.8 ± 5.9°) show a better bias but a lower precision. The lower performance in precision might be associated with the higher diversity of subjects and speeds analyzed in this study. Also, the authors assumed that the pitch and roll angles were similar for every stance phase, hence reducing the inter-steps variability of the system. Koska et al. (2018) used trapezoidal integration of gyroscopic measurements to estimate the orientation of the foot during the stance phase. The authors reported error biases (°) ±95% limits of agreement (°) of −3.1 ± (−7, 3.4) at 10 km/h, −3.8 ± (−7.6, 2.1) at 12 km/h, and −5.9 ± (−11.1, 1.8) at 15 km/h in the estimation of the sagittal plane (i.e., pitch angle) range of motion during stance phase. Although heel-off events were defined using a fixed time window, their observations corroborate with the results of the MSDI method. Shiang et al. (2016) also assumed the presence of a foot-flat period (i.e., pitch angle = 0°) during stance, as for the MSDI method used in the present study, and defined the difference between two local maximums as the strike index. The range of angles (−5°, 27°) reported for the strike index reflect those obtained for θ_JCA_(AC) and θ_MSDI_(AC) parameters in this study. Also, in Altman and Davis (2012), the authors concluded that the foot strike angle, obtained from an optical motion capture system, is an acceptable measure of foot strike pattern, and proposed the following classification limits: rearfoot strike>8°, midfoot strike between −1.6° and 8°, and forefoot strike <1.6. These results, with a midfoot strike classification range of 9.6°, suggest that the JCA method provides an acceptable measure of the pitch angle, with an accuracy of 2.0 ± 5.9° and 0.9 ± 6.7° for the θ_JCA_(AC) and θ_JCA_(IC) parameters, respectively. However, such a conclusion does not hold for the MSDI method (Table 1). A validation study on walking analysis (Bourgeois et al., 2014) reported accuracy and precision of 0.5 ± 2.9° and 3.9 ± 5.8° in the estimation of the pitch angle (θ) at initial and TC, respectively. In comparison, we observed (when the steps were gathered regardless of their trial) a 0.8 ± 5.9° and 17.0 ± 9.0° accuracy and precision for θ_JCA_(IC) and θ_JCA_(TC) parameters. The lower performance may partly be explained by the lower detection accuracy of the initial and TC in running and by the highly dynamic motion of the foot in running generating a greater level of noise during the period of stance.

The results from the Kruskal-Wallis test suggest that neither the bias nor the precision of the θ_JCA_(AC) and θ_JCA_(MS) parameters were significantly affected by the running speed. However, we observed significant differences in the precision of θ_JCA_(IC) and θ_JCA_(TC). This possibly results from the effect speed has on the detection precision and accuracy of IC and TC (Falbriard et al., 2018). Note that there is a performance tradeoff made by the system and associated with the running speed. At low running speeds, the norm of the tangential and centripetal accelerations during the pushing phase is small and therefore decreases the performance of the automatic estimation of ${\vec{r}}_{p\,c}$ in Eqs. 9–13. Conversely, ground contact times are longer at low running speeds (Nummela et al., 2007), increasing the probability of sufficiently long static periods to directly estimate the 3D orientation of the rearfoot segment.

It is essential to bear in mind that the present study was performed on a 0° inclined treadmill. Consequently, the results reported in this document cannot be generalized to uphill and downhill running. Also, we obtained the reference orientation based on markers on the sensors rather than on the shoe; therefore, the protocol constraints such as the lightweight IMU and the IMU fixation are aspects that could affect the detection results. Finally, the present study raises the possibility for the JCA method to be tested on active gait methods other than running (e.g., Nordic walking).

## Conclusion

In this study, we proposed and validated a new method to estimate and correct the orientation drift estimation based on a foot-worn IMU using a two-segment model of the foot for drift removal. The validation compared sagittal and frontal plane angles obtained from an optical motion-tracking system with the estimation based on wearable inertial sensors. We showed that the pitch angle at mid-stance can be estimated with an inter-trial median ± IQR of 0.4 ± 3.8° and an inter-trial precision median ± IQR of 3.0 ± 1.8°. Although running speed can affect the detection performance, the system showed a good agreement with the gold standard optical motion-tracking system. Apart from the short standing period used for the functional calibration, the proposed system is fully plug-and-play. It requires no prior knowledge about the position of the sensors and needs no magnetometer.

## Data Availability Statement

The raw data supporting the conclusions of this article will be made available by the authors, without undue reservation, to any qualified researcher.

## Ethics Statement

The studies involving human participants were reviewed and approved by the CCER-VD. The patients/participants provided their written informed consent to participate in this study.

## Author Contributions

MF, FM, BM, GM, and KA conceptualized the study design and contributed to the analysis and interpretation of the data. MF and FM conducted the data collection. MF designed the algorithms. KA supervised the study. MF drafted the manuscript, and all other authors revised it critically. All authors approved the final version and agreed to be accountable for all aspects of this work.

## Conflict of Interest

BM was employed by the company Gait Up.

The remaining authors declare that the research was conducted in the absence of any commercial or financial relationships that could be construed as a potential conflict of interest.
